# Personalised Online Upper-Limb Physiotherapy for Stroke Survivors during the Inpatient Phase: A Feasibility Study

**DOI:** 10.3390/healthcare11182582

**Published:** 2023-09-19

**Authors:** Abdullah Ibrahim Alhusayni, Eileen Stewart Cowey, Elaine Coulter, Mark Barber, Lorna Paul

**Affiliations:** 1 College of Applied Medical Sciences, Shaqra University, Sahqra 11961, Saudi Arabia; 2 Nursing & Health Care School, University of Glasgow, Scotland G12 8QQ, UK; eileen.cowey@glasgow.ac.uk; 3 School of Health and Life Sciences, Glasgow Caledonian University, Glasgow G4 0BA, UK; elaine.coulter@gcu.ac.uk (E.C.); lorna.paul@gcu.ac.uk (L.P.); 4 NHS Lanarkshire, Scotland G67 1BJ, UK; mark.barber@nhs.scot

**Keywords:** stroke, physiotherapy, telerehabilitation, upper limb, task-specific exercises

## Abstract

Background: After a stroke, inpatients often receive less than the recommended dose of therapy. Telerehabilitation may assist by providing personalised rehabilitation programmes without face-to-face therapy time. This study aimed to evaluate the acceptability and feasibility of an individualised programme of upper-limb rehabilitation that is delivered via an online rehabilitation platform for inpatient stroke survivors. Methods: Stroke survivors were recruited from three stroke units in one NHS Board in Scotland and randomised to the intervention (personalised upper-limb exercise programme delivered via an online physiotherapy platform for four weeks, up to 30 min five times per week, in addition to usual care) or the control group (usual care). The main outcomes are related to recruitment, attrition, adherence and safety. The clinical measures were the Action Research Arm Test, Trunk Impairment Scale and Modified Ashworth Scale. The intervention participants, their carers and physiotherapists completed questionnaires on the acceptability of the intervention. Results: Twenty-six participants, 42% males, were recruited around three weeks post-stroke, on average. There were 13 participants in each group, with a mean age of 69 years (SD of 12) and 67 years (SD of 11) for the control and intervention groups, respectively. Overall, 47% of those screened for eligibility were randomised, and attrition was 23% in the intervention group mainly due to discharge before the end of the intervention. Participants who adhered to their programme (completed more than two-thirds), generally those with an engaged carer, demonstrated a trend toward improved clinical outcomes. Overall, the patients, carers and physiotherapists were positive regarding the intervention. There was a total of five reported adverse events, none of which were related to the study. Conclusion: An upper-limb unsupervised exercise intervention using an online physiotherapy platform for inpatient stroke survivors is feasible, safe and acceptable to patients, carers and physiotherapists. A fully powered RCT is warranted to investigate the clinical- and cost-effectiveness of such interventions for this patient group.

## 1. Introduction

Stroke incidence has been increasing, and in 2019, there were 12.2 million incident cases of stroke and over 101 million people living with stroke globally [1]. There are approximately 1.3 million stroke survivors in the United Kingdom (UK), with a stroke affecting 100,000 people per year in the UK [2]. Stroke care consumes around GBP 26 billion of the UK National Health Service (NHS)’s budget every year [3].

Stroke is one of the leading causes of disability worldwide [1]. Some people with stroke experience long-term motor deficiency, impaired functional activities and decreased participation in activities of daily living [4]. Upper-limb paresis affects 69%, and lower-limb paresis 61%, of stroke survivors in the acute stage [5], and about 50% of stroke survivors still have limited arm function six months post-stroke [6]. A stroke may lead to a loss of active movement and decreased sensation, dexterity and coordination in the affected arm, possibly preventing stroke survivors from undertaking normal everyday activities, such as eating, drinking, dressing and washing [7]. 

Rehabilitation features highly in the top priorities for stroke research, including improving upper-limb function [8]. Rehabilitation after stroke is based on the concept of neuroplasticity, where there is reorganisation (changes in function and structure) of the remaining cortical tissue in response to stimulation through activity [9]. Repetitive task-specific training optimises motor (re)learning and neuroplastic changes in the nervous system, improving patient outcomes [10,11]. High repetitions of task-specific movements that are functionally relevant and meaningful to stroke survivors are required in order to improve the functional recovery after stroke, as suggested by animal studies [12]. The first month after stroke is the peak time for nervous system neuroplasticity [13]; therefore, rehabilitation during this time is important for optimising functional recovery.

There is strong evidence that the dose of stroke rehabilitation is correlated with the degree of functional recovery [11,14,15,16,17]. Recently published national clinical guidelines for stroke in the UK recommend that post-stroke rehabilitation should involve a minimum of 3 h of multidisciplinary therapy, 5 days per week, with the intensity increasing based on the ability of the stroke survivor [18]. However, the dose of therapy received is often lower than these recommendations [19]. Barriers to achieving the recommendations include low staffing levels, increased time required for non-direct patient-related activity (e.g., daily handovers and documentation) and patient-related issues such as not being well enough to take part in therapy [20]. During the hospitalisation period, stroke survivors generally receive their therapy sessions throughout the working week; however, to increase the amount of rehabilitation, a few stroke units offer seven-day services [19]. There is also evidence that inpatient therapy sessions tend to focus on rehabilitation of the lower limbs, highlighting the need for stroke survivors to have extra upper-limb rehabilitation [21,22]. 

Augmenting the current rehabilitation provision and increasing the overall therapy time/dose for stroke survivors have been investigated in a number of studies; however, these studies often involve fully or partially supervised interventions [23,24,25,26,27,28], which do not address the issue of staffing levels and associated costs. Unsupervised, augmented upper-limb rehabilitation involving written sheets of exercises was found to be safe and feasible [29,30] and to improve functional recovery after stroke, with a high level of satisfaction [30,31]. The use of technology tools to augment the current rehabilitation provision is recommended by the recently updated UK National Clinical Guideline for Stroke [18,32].

There is increasing, although sometimes equivocal, evidence of the effectiveness of telerehabilitation in improving clinical outcomes, including upper-limb functions, after stroke [33,34,35,36]. Most studies focus on providing home-based telerehabilitation; however, telerehabilitation programmes could be a cost-effective approach if implemented in the inpatient setting to increase the therapy dose by encouraging patients to exercise without therapy times, particularly in the evenings and weekends, with the support of carers, if required. 

The aim of this study was to evaluate the acceptability and feasibility of an individualised four-week programme of upper-limb rehabilitation, delivered via an online rehabilitation platform, for inpatient stroke survivors. 

## 2. Materials and Methods

### 2.1. Ethical Considerations

Prior to the study, an existing online rehabilitation platform “www.giraffehealth.com accessed on 1 August 2020)” was adapted for use in a stroke population through co-production focus groups. The study was approved by the West of Scotland Research Ethics Committee (REC Ref 18/WS/0101), and Research and Development (R&D) approval was given by NHS Lanarkshire (R&D Ref L18047). The trial was registered on ClinicalTrials.gov (Ref. NCT03666702). Through the platform, the patient received a personalised video-based exercise programme, a stroke-specific advice page (including an aphasia version) and an exercise diary to complete if able. Participants were recruited from the three stroke units within NHS Lanarkshire, Scotland, UK. There were three groups of participants: stroke survivors, their carers and the physiotherapists. The study commenced on September 2018 and ended in August 2019. The study followed the Consolidated Standards of Reporting Trials (CONSORT) reporting guidelines [37].

The study aimed to recruit 30 stroke survivors—15 to the intervention group and 15 to the control group. This figure was felt to be feasible based on a 12-month recruitment period.

### 2.2. Inclusion/Exclusion Criteria

Stroke survivors were invited to take part if they were over 18 years old, had moderate-to-severe upper-limb functional limitation due to stroke (score 0–39 in the Action Research Arm Test (ARAT)) [38], were in the stroke unit with their first stroke, were able to sit in a chair or bed, could use a computer or tablet with or without help from carers, could understand English and we able to provide informed written consent. Stroke survivors were excluded if they had significant comorbidity, which would preclude them from taking part in an exercise programme; had moderate-to-severe cognitive impairment (<25 in the Mini Mental State Examination (MMSE) [39]; or they were participating in another interventional research study. Initially, anyone with a shoulder subluxation was excluded; however, it became apparent that almost all potential participants had some degree of shoulder subluxation, so a substantial amendment request was successfully made to the Ethics Committee (REC Ref AM01) to exclude only stroke survivors with shoulder subluxation greater than grade 3 (more than 1½ fingerbreadth gap) [40]. 

### 2.3. Data Collection

Stroke survivors were informed of the study by the physiotherapists in the stroke unit. They were given a participant information sheet (PIS) and were advised to contact the research team or physiotherapists if they would like to take part in the study. Aphasia-friendly versions of the consent form and PIS were available if required. 

Participants who expressed an interest were assessed for eligibility before completing a consent form. Age, sex, time since stroke, ethnicity, level of education and stroke severity (National Institutes of Health Stroke Scale (NIHSS) [41]) were recorded. The NIHSS scale is scored from 0 to 42, with higher scores indicating higher stroke severity. In addition, social history and walking status before stroke, previous information technology (IT) use (computers/tablets/phones), medical history (stroke risk factors, comorbid conditions and previous transient ischemic attack (TIA)), stroke type and location (cortical, subcortical, midbrain or brainstem) were noted. Then, a baseline (initial) assessment was completed (see below). 

Participants were then randomised. A set of sequential numbers (1–30) was generated by the researcher. Each number was placed in a separate envelope, and the envelopes were shuffled. Participants were randomised by choosing one of the envelopes: envelopes with odd numbers allocated participants to the intervention group, and even numbers allocated participants to the control group. The researcher (AA) was also the assessor and therefore was not blind to the group allocation.

Intervention: The intervention was developed and reported in accordance with the template for intervention description and replication (TIDieR) guidelines [42]. All stroke participants were given a leaflet from Chest Heart & Stroke Scotland about exercise and physical activity. The control group received usual physiotherapy care only. This was provided one-to one by physiotherapists and/or assistant physiotherapists for 4 or 5 sessions per week, with each lasting up to 45 min. The number, duration and content of all physiotherapy sessions were recorded by physiotherapists and occupational therapists for both groups, using forms developed for the study. Completed TIDieR checklist for the online physiotherapy programme has been uploaded it as Supporting Material. 

Stroke survivors in the intervention group received a progressive, individualised four-week upper-limb physiotherapy intervention delivered via the Giraffe Healthcare online rehabilitation platform “www.giraffehealth.com (accessed on 1 August 2020)”, in addition to usual care. The individualised programme of upper-limb and trunk exercises was prescribed by the participant’s physiotherapist and was based on clinical assessment, their goals and their level of upper-limb function. The exercise dose (duration, frequency and intensity) was based on the participant’s level of functional ability, aiming to achieve 30 min of exercise, five sessions per week (including weekends), in addition to their usual physiotherapy care. 

Participants in the intervention group received an explanation of their upper-limb programme and how to use the website. Physiotherapists reviewed their progress once a week and made any necessary changes to their programme. Participants were able to contact the research team during the study to ask any questions or to request a change in their programme. If participants had communication difficulties, where appropriate, an explanation of the participant’s upper-limb programme and how to access the website was provided to their carers, who then supported them to undertake their exercise programme. In addition, an aphasia-friendly version of the advice section on the website was available. Stroke survivors used their own tablets/laptop to access their programme; however, if they did not have an internet-enabled device, they were provided with a tablet and/or internet access for the duration of the study. 

### 2.4. Outcome Measures 

The main outcome measures related to the feasibility of the study are as follows:

(1) Recruitment: number of stroke survivors who fulfilled the inclusion criteria and the number who agreed to take part.
(2) Attrition: number of participants who dropped out.
(3) Adherence: number of completed exercise diaries per week over the intervention period (maximum of 20 sessions, 5 sessions a week for 4 weeks) expressed as a percentage. Participants completing at least two-thirds of their prescribed sessions (13 sessions) were considered adherent [43].
(4) Safety: number of related adverse and serious adverse events.

Clinical assessments were carried out by a researcher (AA) who was not blinded to the group allocation. The following assessments were undertaken in both groups of stroke survivors at baseline (week 0) and post-intervention (week 5). The Action Research Arm Test (ARAT) is a valid and reliable tool for stroke survivors and measures arm function in 19 items grouped into the following subscales: grasp (6 items), pinch (6 items), grip (4 items) and gross motor (3 items) [44,45]. Scores range from 0 to 57 (maximum) [38]. The Minimal Clinically Important Difference (MCID) for the ARAT in stroke survivors is an increase of 5.7 points [38]. The Trunk Impairment Scale (TIS) consists of 17 items for assessing the level of motor impairment of the trunk [46]. The scores range from 0 to 23, with lower scores indicating high levels of trunk impairment. The TIS is a valid and reliable scale for stroke [47]. The Modified Ashworth Scale (MAS) is also valid and reliable in the stroke population [48]. The scores range from 0 to 4, with higher scores indicating higher muscle tone. The spasticity of the shoulder adductor, elbow flexor, wrist flexor and finger flexor muscle groups was examined using the guidelines of [49]. The MAS is most reliable for assessing the elbow and wrist; therefore, it was appropriate for assessing upper-limb spasticity in this study [50,51]. In addition, the views of the stroke survivors in the intervention group and their carer (if relevant), as well as the physiotherapists, were sought using three versions of a developed questionnaire. All questionnaires were administered by the researcher (AA), face-to-face, to all participants. The questionnaires were piloted in two stages to make sure that important topics were covered fully, questions and instructions were clear and none of the questions had been objected to. The questionnaire for stroke survivors included sections on the exercise programme, any help need to complete the programme, the frequency with which it was completed and views on the Giraffe platform. Generally, closed questions were used, but free-text space was included, so participants could expand their responses. Stroke survivors’ questionnaire has been uploaded as Supporting Material.

Carers: It was not mandatory that all stroke participants allocated to the intervention group had a carer who agreed to participate. Carers were defined as the person most likely to help the participant complete his/her exercise programme. The physiotherapists in the stroke unit informed the carers about the study. Once the stroke survivor was allocated to the intervention group, the carer was provided with a PIS. Carers were eligible to take part if they were over 18 years, able to communicate in English and able to support the stroke survivor with his/her online exercise programme. Following consent, demographic information was recorded: age, gender, relationship to stroke survivor, occupation, ethnicity, level of education, previous use of IT and general health status. The carer’s questionnaire covered similar areas to the stroke survivors but with questions related to the support they gave during the intervention period. Physiotherapist’s questionnaire has been uploaded as Supporting Material.

Physiotherapists: To be included, physiotherapists had to have delivered the intervention to at least two stroke survivors. Following consent, demographic information was recorded: age, gender, physiotherapist or assistant physiotherapist, level of education, previous use of IT and time working in stroke rehabilitation. The physiotherapist’s questionnaire gathered views on the exercise programme, the Giraffe platform and any challenges experienced. Physiotherapist’s questionnaire has been uploaded as Supporting Material. 

### 2.5. Analysis

Quantitative data were analysed using the Statistical Package for the Social Sciences (SPSS) version 25. Data are presented as means with standard deviations (SDs), or medians with ranges (where appropriate) for continuous variables and numbers with percentages for ordinal and categorical variables. 

For the clinical outcome measures (ARAT, TIS and MAS), medians with range baseline assessments and post-intervention assessments were calculated for each group.

For the feedback questionnaires, the frequencies and percentages were calculated to summarise the findings of ordinal and categorical variables and quotes from the free text sections used to expand upon the responses. 

## 3. Results

### 3.1. Baseline Demographic and Stroke Characteristics

Twenty-six participants were recruited to the study—thirteen in each group (Table 1). The cohort comprised 11 males (42.3%) and 15 females (57.7%) and was allocated evenly to both groups. The mean age was 69 years (standard deviation (SD) of 12) and 67 years (SD of 11) for the control and intervention groups, respectively. Participants were recruited three weeks after stroke, on average. Twelve participants (46.1%) previously used computers daily, and one participant (3.8%) used computers only with the assistance of a relative. 

Most participants (88.4%) were able to walk independently before their stroke, and only one participant from each group (7.7%) was able to walk independently after the stroke. The NIHSS scores were slightly higher for the control group than the intervention group (Table 1).

All had their strokes confirmed by imaging (most commonly CT scan), and five participants from each group received thrombolysis/reperfusion therapy. More information about stroke characteristics is provided in Table 2. 

### 3.2. Outcome Measures 

**Figure 1:**
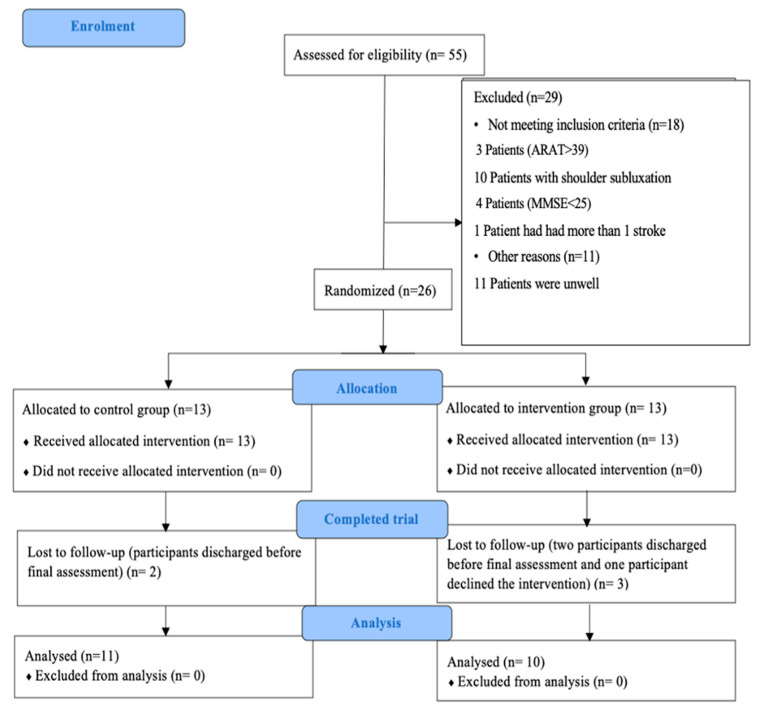
CONSORT flow diagram for recruitment of the participants.

Feasibility Outcomes: Overall, 26 participants were recruited, 4 short of the target of 30 participants. Over the 12-month period of recruitment, 55 participants were assessed for eligibility, of which 29 did not fulfil the criteria, most commonly due to shoulder subluxation, and another 11 participants were generally too unwell to take part. Twenty-six participants were recruited equally into the intervention and control group. Both groups received the planned intervention (Figure 1). Around 5–6 participants were screened per month, and 47% of those were randomised. Ten people from the intervention group and eleven people from the control completed a post-intervention assessment. Thus, attrition was 23% in the intervention and 15% in the control group. 

Of the ten participants who completed the intervention period, seven (70%) logged into the online rehabilitation platform and completed at least one complete exercise session. Of the remaining three participants, two did not log into the platform at all, and one participant logged into the platform and performed some exercises but did not complete a full exercise session.

Out of the seven participants who undertook some exercise, five participants were adherent, as they performed more than two-thirds of their prescribed intervention (Table 3). Overall, participants in the intervention group completed 3.7 exercise sessions per week, while those who were considered adherent completed 5.3 sessions per week, on average. 

The number of prescribed exercises for the nonadherent participants was 3–5 exercises per session, and for the adherent participants, 3–10 exercises per session. Furthermore, there were no specific days of the week (weekdays or weekends) that the participants chose to undertake their prescribed exercises. 

Participant safety: There were five recorded adverse events, namely shoulder pain (n = 2), fatigue (n = 1) and fall (n = 2), none of which were deemed to be related to the study intervention. 

Clinical outcome measures: For the clinical measures, in the intervention group, the median ARAT was 0 (0–36) at baseline, with seven participants scoring 0. This increased to 11 (0–57) post-intervention, with 2 participants scoring 0. In the control group, the median ARAT at baseline was 0 (0–21), with eight scoring 0, and this increased to 11 (0–44) post-intervention, with four participants scoring 0. The ARAT scores for 6 out of those 15 participants who had a score of 0 did not change (two intervention group and four control group), while all of those with an ARAT of 1 or more improved over the intervention period, whether in the control or intervention group. In the intervention group, 8 out of 10 participants improved by more than the MCID (5.7 points) in the ARAT, while in the control group, 6 out of 11 participants showed clinically significant improvements. 

There was an overall trend for both intervention and control groups toward an increase in TIS. In the intervention group, the median TIS was 16 (4–21) at baseline and increased to 17 (10–23) post-intervention. In the control group, the median TIS at baseline was 13 (0–19), increasing to 17 (7–21) post-intervention period. The median difference between the two groups in TIS score was similar.

An assessment of muscle spasticity (MAS) was performed for the shoulder adductor, elbow flexor, wrist flexor and finger flexor muscle groups. There were no notable trends for either the intervention or control group in any of the assessed muscle groups (Table 4).

Stroke survivor’s questionnaire: All the participants who completed the intervention responded to the questionnaire (n = 10). Three did not do any of their exercise programmes; therefore, they did not provide feedback on the study intervention. The main reason why participants did not undertake any exercise was *“no [internet] signal at the ward” (Stroke Survivors 27 and 3)*. Four participants reported doing their exercises from memory, without accessing their exercise programmes; therefore, the exercise diary provided an underestimation of completed exercise sessions for those participants.

In general, the participants found the exercise programme easy to understand and beneficial and did not believe that it increased their level of fatigue, with most indicating that they would be happy to use the platform again in the future. Overall, the participants expressed positive views about using the platform to do their exercise, and the majority had no difficulties learning how to use it (Table 5).

Three participants reported using the online platform 3–5 times per week, one participant reported 7 times per week, and three participants reported 14 times per week. The majority of participants (71.4%, n = 5) reported spending less than 30 min per session, and 28.6% (n = 2) spent up to an hour per session. Six participants reported requesting help from carers to do their exercise programme. None of the participants asked staff for help. Four stroke survivors explained that they needed help from their partner/relative to provide motivation—*“Encourage me to do my exercise” (Stroke Survivor 17)*—and physically supporting the weaker part of the body: *“Help me with my weak hand and the shoulder” (Stroke Survivor 5).*

Five participants indicated that practising the exercises was easy for them because the website was clear and easy to use.

*“It was self-explanatory, you just watch and listen, it was simple” (Stroke Survivor 10).*

Carer’s questionnaire: Five carers were recruited, two males and three females, with a mean age of 36.8 (SD 11) years. Two of the stroke survivors had two carers helping them with the exercises and who responded to the questionnaires separately. Three had a college degree, one had a postgraduate degree and the other had other qualifications. All indicated that they use computers daily, and their professional careers varied from information technology officer to housewife.

All strongly agreed that it was easy to help their partner/relative to do the exercises, that the exercises were clear and understandable and that they would be happy to use the online platform to help their partner/relative in their future rehabilitation exercises. All strongly agreed that learning to use the platform was easy. Three participants (60%) indicated that they helped their partner/family member 7 times per week, and two participants (40%) indicated 3–5 times per week. They stated that they helped the stroke survivor to access and perform the exercise programmes. Similar to stroke-survivor participants, none of the carers asked the staff for help.

Physiotherapist’s questionnaire: Five physiotherapists, one male and four females, with a mean age of 35.4 (SD 7.7) years, were recruited. All identified as computer literate and had worked in stroke rehabilitation for a mean of 4.4 (SD 3.6) years. Four monitored stroke survivors’ exercise programmes once per week, and one monitored the programmes twice per week. 

Overall, the responses were positive. The majority of physiotherapists found the exercises to be clear and well explained, and they expressed that they would be happy to use the website again. The participants showed some concerns about the effect of monitoring the online intervention on their workload (Table 6).

The physiotherapists provided details about the advantages/disadvantages of the online exercise programme, with advantages such as involving the patients in their rehabilitation.

*“Helps patients to feel involved and control of their rehabilitation and influence in their mental health” (Physiotherapist 2).*

They also felt that the platform provided opportunity for carers to support stroke survivors in their rehabilitation journeys and to encourage additional exercise out with usual care to help promote recovery:

*“Good idea to have such a website for families to assist with the exercise” (Physiotherapist 9).*

*“Patients use their spare time to do more exercise beneficially for recovery” (Physiotherapist 2).*

Finally, the simplicity of the website made things easier for stroke survivors and physiotherapists:

*“Exercise very well described and useful to have videos of the specific exercise” (Physiotherapist 9).*

In terms of disadvantages, the physiotherapists highlighted the limited choice of upper-limb exercises on the platform. However, some of the main challenges were technological, relating to poor internet connection in the hospitals and the fact that the website worked on the Chrome browser but the NHS default at the time was Internet Explorer. One physiotherapist highlighted that patients often did the exercise from memory without logging into the website.

*“Not all patients managed to use tablet themselves” some patients remembered to exercise and then did not log in to do them each time” (Physiotherapist 7).*

Finally, there was a recognition that online exercise might not be suitable for all stroke survivors, including older adults.

*“Limited for patients with cognitive impairment” (Physiotherapist 2).*

*“Not suitable for all elderly people” (Physiotherapist 6).*

*“Many patients found it difficult to use and felt too tired to do as additional therapy work alongside daytime therapy” (Physiotherapist 9).*

## 4. Discussion

Overall, this study demonstrated that an unsupervised upper-limb rehabilitation programme delivered via an online platform “www.giraffehealth.com (accessed on 1 August 2020)” was feasible and acceptable to stroke survivors, their carers and their physiotherapists at the inpatient stage of their rehabilitation, although a few issues need to be considered for a future fully powered randomised controlled trial. 

Over the 12-month period of the study, 5–6 patients were screened per month in this single-centred study, 47% of which were randomised. Therefore, subsequent studies should aim to screen at least double the number of participants needed for the study to ensure recruitment figures are achieved, assuming similar recruitment figures in different clinical sites.

One of the main reasons for not being recruited was shoulder subluxation (n = 11), as per the initial inclusion/exclusion criteria (n = 10). Amending the inclusion criteria to only exclude participants with shoulder subluxation greater than grade 3 [40] significantly improved recruitment and did not lead to adverse events; therefore, the amended exclusion should be used in any future study. 

Including participants with severely limited arm functions (ARAT = 0) resulted in the delivery of passive exercises rather than functional-task-specific/oriented exercises; however, half of participants with an ARAT score of 0 (7 out of 13) improved their score after the intervention, indicating the potential for recovery in patients even with severely limited arm function. The presence of voluntary movement in shoulder abduction and finger extension within three days of stroke can be a predictor for full recovery of upper-limb function for up to 60% of stroke survivors [52]. Future studies might consider stratifying participants based on their ARAT score at randomisation to better understand the potential effect of the study intervention on stroke survivors with different levels of upper-limb impairment. 

The overall level of participant attrition was 19% (23% in the intervention group and 15% in the control group). The main reason for attrition was the patient being discharged from the stroke unit before the end of the study period. Participants were randomised on average three weeks from their stroke; thus, future studies may aim to reduce this period if the participant is medically stable, especially given the increase in early supported discharge (ESD) from stroke units. However, based on these figures, a sample size calculation for a future fully powered randomized controlled trial (RCT) should allow for at least 19% attrition. 

There were five reported adverse events during the study period. Although adverse events are expected in an acute–subacute stroke population, none of the recorded adverse events were deemed directly related to the study; therefore, it can be concluded that the intervention showed no signal for harm in the current study. 

Adherence to telehealth interventions to improve physical function has been poorly reported in previous studies [33]. In terms of adherence, seven out of ten participants (70%) who completed the intervention period logged into their exercise programme and completed at least one exercise session; five were adherent, as they completed at least two-thirds of their prescribed sessions (mean of 5.3 sessions/week). This figure is comparable with a previous study of unsupervised upper-limb exercise programmes for hospitalised stroke patients using printed sheets of exercises, where participants were asked to complete the augmented intervention 1 h a day, 6 days a week, for 4 weeks and completed, on average, 3 h per week and 4.8 days per week [30]. The evaluation questionnaires in the present study, however, highlighted that four participants completed their exercise programme without logging; thus, the adherence figures in the present study may be an under-estimate. Participants using the platform in future studies should be reminded to record their exercises to provide more accurate data on adherence to the intervention. 

Three participants in the present study did not complete any exercise sessions, which is similar to another study of online exercise in the management of type 2 diabetes, which found that 28% of participants never logged into the website [53]. The present study is the first study to use an online exercise platform during the inpatient stage of stroke rehabilitation; thus, the context is different from previous studies in stroke or other conditions but demonstrates that additional measures may be required to support some participants to engage with online interventions. 

The main reason for participants not using the online exercise platform was poor Wi-Fi signal in the ward. Future studies should ensure that there is stable internet connection at the study site(s), but this also highlights the lack of infrastructure in some inpatient NHS facilities. 

Many stroke-survivor participants sought help from their carers to access the website and/or perform their exercises, and most of the participants who were adherent to the augmented programme had a carer to assist them. Thus, patients who have carer support may be the ones who are most likely to benefit from this programme. The Early Versus Later Augmented Arm Physiotherapy (EVERLAP) trial, which involved up to 27 additional hours of upper-limb exercise (both supervised and unsupervised) over six weeks at time points up to nine weeks after stroke [54], also reported that the presence of carers to support stroke survivors facilitated their engagement with the unsupervised sessions [54].

There was also a trend toward better adherence in those with a greater number of exercises in their programmes, but the optimum number of exercises to improve adherence requires further investigation. Importantly, however, the intervention was not found to be a burden for participants in addition to their usual care/therapy, with the exception of one therapist who reported that as an issue. 

In terms of the clinical outcomes assessments, both groups improved, thus demonstrating recovery with usual care post-stroke. The overall changes in the ARAT scores in this feasibility study were in favour of participants in the intervention group, as more participants in this group had improvement exceeding the MCID (5.7 points in scores). Although this provides some indication of the likely effect of the augmented intervention on upper-limb function, this study was not fully powered to detect differences between groups. The overall completeness of ARAT scores in this study was good, with missing scores only from those discharged before the final assessment. This would suggest that the ARAT is a viable primary outcome measure for any further study on the efficacy of upper-limb interventions for hospitalised stroke survivors. 

In terms of spasticity, there was no indication of an increase in muscle tone in either group, as demonstrated by an increase in MAS. Previous studies of fully supervised augmented upper-limb exercises in inpatient stroke survivors similarly did not find any statistically significant changes across groups [45,55,56]. Given the time taken to complete the MAS and the low levels of spasticity in this stroke population, it may not be appropriate to include the MAS in a future study.

The TIS was assessed in this study because having a good trunk function is essential to be able to control upper-limb function [57]. A previous study also found telerehabilitation to be a feasible modality to deliver unsupervised augmented trunk exercises for stroke survivors in the chronic stage [58]. Given that trunk exercises can positively affect functional abilities and that this effect is at its peak in the acute stage after stroke [59], the TIS would seem an appropriate outcome to be included in any subsequent study.

There were only three clinical outcomes in this feasibility study; other domains may be relevant. Future studies should consider employing the recommended outcome measures for post-stroke arm-rehabilitation trials [60].

The ease of use of the technology, attractive user interface and personalisation of the programme are factors reported to improve adherence and user satisfaction with telerehabilitation for upper-limb disability, including stroke [61]. The results from the questionnaires demonstrated that stroke survivors, carers and physiotherapists thought that the exercise programme delivered through the online physiotherapy platform was clear, easy to use and helpful and that they would be keen to use it again in the future. The stroke survivors were happy with the exercise programme, and although one physiotherapist suggested that the programme increased patient fatigue, the stroke survivors did not report an increase in fatigue. 

The physiotherapists who participated in the study stated that the process of setting up new patient exercise programmes on the website was easy and was not considered an imposition on their daily workload. However, the platform was not compatible with the browser used by the clinical site at the time, thus causing some issues, together with a perceived limited number of upper-limb exercises on the platform. The NHS now uses the Chrome browser more widely, and there has been a significant increase in the number of upper-limb exercises on the Giraffe platform so that these issues will not affect future studies. 

Another study has investigated telerehabilitation in acute stroke. In the CARE4STROKE trial, carers supported stroke survivors with personalised exercises through an app, aiming for an additional 150 min of exercise per week for 12 weeks. There was no difference in length of hospital stay or mobility; however, there was a reduction in patient anxiety and caregiver depression [62]. It transpired that both groups undertook a similar number of minutes of exercise, and this may explain the similarity in clinical outcomes between groups. As with our trial, this study used technology to deliver augmented rehabilitation and recruited stroke survivors during their hospital period. However, while this study used CARE4STROKE application, our trial used the Giraffe platform to deliver augmented intervention. In addition, while the augmented intervention in this other study aimed to increase the intensity of practice and facilitate ESD, our trial aimed to improve upper-limb function. 

The results of this study suggest that telerehabilitation, using an online exercise platform, has the potential to significantly increase the overall dose of therapy for inpatient stroke survivors, without adding significantly to the workload of the therapist. This may address the reported subtherapeutic dose of therapy often reported after a stroke [18,19,63] and, although further research is required may, in turn, improve clinical outcomes. This approach, however, may not be suitable for every stroke survivor, e.g., those with low health literacy or IT skills. We developed aphasia-friendly versions of the PIS, consent form and advice page to support the inclusion of people with aphasia; however, those resources were used by only one participant. 

This study, however, had some limitations. The assessor and the participants were not blind to group allocation, which may introduce bias. Although blinding clinical physiotherapists and participants to group allocation is difficult, any future study should ensure that the assessor is blind to the group allocation. In terms of the dose of the augmented intervention, the sets and repetitions of exercises to complete can be extracted from the Giraffe platform, but not the time taken. In addition, participants often completed exercise sessions but did not log these on the platform. Future studies should more accurately calculate the exercise dose to evaluate the dose effect and ensure that all exercise sessions are logged. The content of usual care was not recorded for either group. Therapists were asked to complete a form of upper-limb exercises included in “usual care” sessions, as well as the duration of these exercises. However, the majority of these forms were not completed, and alternative methods to record the content of therapy sessions is required for any future RCT. Any changes in medications, such as anti-spasticity drugs, were not recorded, but this would be required in a future study.

## 5. Conclusions

Delivering an unsupervised augmented upper-limb exercise intervention using an online physiotherapy platform for inpatient stroke survivors is feasible, safe and acceptable to patients, carers and physiotherapists. There were a number of factors, such as Wi-Fi access, which adversely affected adherence to the intervention, but adherence was facilitated if a carer supported the participant with his/her exercise. A fully powered RCT is warranted to investigate if this approach could address the subtherapeutic dose of therapy often reported after stroke, as well as the clinical and cost-effectiveness of the intervention for inpatient stroke survivors. However, further consideration of both the dose of therapy and the outcome measures used is required.

## Figures and Tables

**Table 1:** Baseline participants’ characteristics.

Categories	Control Group (n = 13)	Intervention Group (n = 13)
Age: mean ± SD	69 ± 12	66.8 ± 11
Gender:		
Male	5 (38.5%)	6 (46.2%)
Female	8 (61.5%)	7 (53.8%)
Ethnicity:		
White British	12 (92.3%)	12 (92.3%)
Another ethnicity	1 (7.7%)	1 (7.7%)
Educational level (highest degree):		
Secondary school	7 (53.8%)	9 (69.2%)
Primary school	1 (7.7%)	0 (0%)
College	5 (38.5%)	3 (23.1%)
University (postgraduate)	0 (0%)	1 (7.7%)
Living alone	8 (61.5%)	3 (23.1%)
Living in own home	10 (76.9%)	8 (61.5%)
National Institutes of Health Stroke Scale ^1^ Mean ± standard deviation	9.6 ± 6.3	6 ± 2
Action Research Arm Test ^2^—affected side median (range)	0 (0–21)	0 (0–36)

^1^ National Institutes of Health Stroke Scale: 0 = no symptoms, and 42 = severe stroke. ^2^ ARAT total scores: 0 = inability to perform any part of the test, and 57 = normal arm performance.

**Table 2:** Stroke characteristics.

Categories	Control Group (n = 13)	Intervention Group (n = 13)
Stroke risk factors:		
Diabetes	2 (15.4%)	2 (15.4%)
Hypertension	4 (30.8%)	8 (61.5%)
Smoking	5 (38.5%)	3 (23.1%)
Alcohol consumption	5 (38.5%)	4 (30.8%)
Coronary artery disease	4 (30.8%)	2 (15.4%)
Atrial fibrillation	2 (15.4%)	1 (7.7%)
Hyperlipidaemia	0 (0%)	3 (23.1%)
Previous transient ischaemic attack (TIA)	2 (15.4%)	3 (23.1%)
Stroke type:		
Ischaemic	8 (61.5%)	9 (69.2%)
Haemorrhage	5 (38.5%)	4 (30.8%)
Stroke subtype:		
Lacunar	1 (7.7%)	4 (30.8%)
Large artery	5 (38.5%)	3 (23.1%)
Undetermined	6 (46.2%)	6 (46.2%)
Missing data	1 (7.7%)	0 (0%)
Stroke location:		
Cortical (internal capsule)	2 (15.4%)	4 (30.8%)
Cortical (middle cerebral artery)	5 (38.5%)	0 (0%)
Cortical (frontal lobe)	1 (7.7%)	3 (23.1%)
Subcortical (thalamus)	1 (7.7%)	0 (0%)
Subcortical (basal ganglia)	1 (7.7%)	3 (23.1%)
Midbrain (medulla)	1 (7.7%)	0 (0%)
Brainstem	0 (0%)	1 (7.7%)
Missing data	2 (15.4%)	2 (15.4%)

**Table 3:** Usage of the online physiotherapy platform and adherence to the physiotherapy programme.

Participant ID	Number of Prescribed Exercises	Number of Completed Sessions	* Percentage of Completed Exercise Diaries	Mean Number of Completed Sessions per Week
5	6–10 exercises	60	280%	14
10	4–5 exercises	19	158%	7.4
17	3 exercises	20	93%	4.7
11	3 exercises	18	84%	4.2
13	4 exercises	9	74%	3.7
28	3 exercises	4	32%	1.7
30	5 exercises	3	24%	1.2
35	6 exercises	0	0%	0
27	5 exercises	Never used the website	0%	0
3	5 exercises	Never used the website	0%	0

* Percentage of completed exercise diaries was calculated using the following formula: (no. of completed programmes/prescribed exercise programmes (5 sessions a week).

**Table 4:** Clinical outcomes at baseline and post-intervention.

Clinical Outcome Measures	Intervention Baseline (n = 13)	Control Baseline (n = 13)	Intervention Post-Intervention (n = 10)	Control Post-Intervention (n = 11)
ARAT				
Median (IQR)	0 (0–36)	0 (0–21)	11 (0–57)	11 (0–44)
TIS				
Median (IQR)	16 (4–21)	13 (0–19)	17 (10–23)	17 (7–21)
MAS (shoulder adductor)				
Median (IQR)	1 (0–2)	2 (0–3)	1 (0–2)	2 (0–3)
MAS (elbow flexor)	0 (0–2)	2 (0–3)	1 (0–3)	2 (0–2)
Median (IQR)	0	0	3	2
MAS (wrist flexor)				
Median (IQR)	1 (0–2)	1 (0–3)	1 (0–2)	1 (0–2)
MAS (fingers flexor)				
Median (IQR)	1 (0–3)	1 (0–4)	1 (0–2)	2 (0–4)

**Table 5:** Participants’ feedback on the intervention and the online platform.

Statement	Strongly Agree	Moderately Agree	Neither Agree nor Disagree	Moderately Disagree	Strongly Disagree
	Frequency				
I feel I benefited from the exercise programme.	5	2			
The exercises were clear and understandable.	5	2			
The exercise programme did not increase my fatigue (tiredness).	5		2		
It was easy to contact the physios to make changes to my exercise programme.	3		3		1
I was happy with the length of time it took for the study assessments.	3	2	2		
I would be happy to do exercises using this website again in the future.	6	1			
Doing my exercises through the website gave me the chance to choose when to exercise.	6		1		
Doing my exercises through the website gave me the feeling of being independent in exercising.	6	1			
Learning to use the website for my exercises was easy for me.	5		2		

**Table 6:** Physiotherapists’ responses to the questionnaire.

Statement	Strongly Agree	Moderately Agree	Neither Agree nor Disagree	Moderately Disagree	Strongly Disagree
	Frequency				
I think the stroke survivors benefited from the exercise programme.	2	2			1
Monitoring the augmented programme did not impose on my day-to-day care of the patients.	1	1	1	2	
The exercises were clear and understandable to the stroke patients.	1	3		1	
I would be happy to provide exercises using this website again in the future.	3	1	1		
Learning to provide exercises using the website was easy for me.	1	1	2	1	
The procedure of signing the stroke survivors up to the website was straightforward.	1	3	1		
The procedure of setting the treatment plan up was straightforward.	1	3		1	

## Data Availability

The datasets used and/or analysed during the current study are available from the corresponding author upon reasonable request.

## Supplementary Materials

The following supporting information can be downloaded at: https://www.mdpi.com/article/10.3390/healthcare11182582/s1, Completed TIDieR checklist for the online physiotherapy programme, physiotherapist’s questionnaire, Stroke survivors’ questionnaire and Carers’ questionnaire.
