# Supplementary material for: Personalised Online Upper-Limb Physiotherapy for Stroke Survivors during the Inpatient Phase: A Feasibility Study
Source: Healthcare (Basel). 2023 Sep 19;11(18):2582. doi: 10.3390/healthcare11182582 (PMC10531470; doi:10.3390/healthcare11182582)
Supplement: Supplementary file 1 [file healthcare-11-02582-s001.zip › Carers' questionnaire.pdf]

Please answer the following questions and add your comments, if appropriate:

**1.** Did you help your partner/relative to do any of his/her exercise programme?	Yes [0]◯ No[1] ◯
**If you answered 'Yes', please go straight to Question 2.**<br><br>**If you answered 'No', could you please let us know why that was?**<br><br>Enter your comments here:<br><br>**(Thanks for your feedback – there is no need to answer further questions)**	

Section 1: Evaluation of the augmented exercise programme:

Question number	Question	Please circle one answer				
**2.**	It was easy for me to help my partner/relative to do the exercises.	Strongly disagree [0]	Moderately disagree [1]	Neither agree or disagree [2]	Moderately agree [3]	Strongly agree [4]
**3.**	The exercises were clear and understandable.	Strongly disagree [0]	Moderately disagree [1]	Neither agree or disagree [2]	Moderately agree [3]	Strongly agree [4]
**4.**	I would be happy to use this website to help my relative/partner exercising again in the future.	Strongly disagree [0]	Moderately disagree [1]	Neither agree or disagree [2]	Moderately agree [3]	Strongly agree [4]

Section 2: Evaluation of the website:

Question number	Question	Please circle one answer				
**5.**	Learning to use the website for my partner/relative's exercises was easy for me.	Strongly disagree [0]	Moderately disagree [1]	Neither agree or disagree [2]	Moderately agree [3]	Strongly agree [4]

Section 3: please answer the following questions and add your comments, if appropriate:

6. How often did you help your partner/relative to do his/her exercise programme?

Once per week [0] ◯ Twice per week [1] ◯ 3-5 times per week [2] ◯ Other [3], please specify:…………

What did you help him/her with?

7. Did you ask staff to help your partner/relative doing his/her exercise programme? Yes [0] ◯ No [1] ◯ **If yes, please answer the following questions:**

How often? Once per week [0] ◯ Twice per week [1] ◯ 3-5 times per week [2] ◯ Other [3], please specify:…………

Who did you ask? Nursing staff [0] ◯ Physiotherapy staff [1]◯ Other [2], please specify:…………

What did you need the staff to help your partner/relative with?

8. How difficult/easy was helping your partner/relative to exercise using the website without supervision?

Easy [0] ◯ Neither easy nor difficult [1] ◯ Difficult [2] ◯

Please provide details. For example, did you worry about helping your partner/relative to exercise properly or knowing how many exercises your partner/relative needed to do?

Please tell us about things that went well? What was difficult (if anything)?
