# Supplementary material for: Personalised Online Upper-Limb Physiotherapy for Stroke Survivors during the Inpatient Phase: A Feasibility Study
Source: Healthcare (Basel). 2023 Sep 19;11(18):2582. doi: 10.3390/healthcare11182582 (PMC10531470; doi:10.3390/healthcare11182582)
Supplement: Supplementary file 1 [file healthcare-11-02582-s001.zip › Physiotherapists' questionnaire.pdf]

Section 1: Evaluation of the augmented exercise programme:

Question number	Question	Please circle one answer				
**1.**	I think the stroke survivors benefited from the exercise programme.	Strongly disagree [0]	Moderately disagree [1]	Neither agree or disagree [2]	Moderately agree [3]	Strongly agree [4]
**2.**	Monitoring the augmented programme did not impose on my day to day care of the patients.	Strongly disagree [0]	Moderately disagree [1]	Neither agree or disagree [2]	Moderately agree [3]	Strongly agree [4]
**3.**	The exercises were clear and understandable to the stroke patients.	Strongly disagree [0]	Moderately disagree [1]	Neither agree or disagree [2]	Moderately agree [3]	Strongly agree [4]
**4.**	I would be happy to provide exercises using this website again in the future.	Strongly disagree [0]	Moderately disagree [1]	Neither agree or disagree [2]	Moderately agree [3]	Strongly agree [4]

Section 2: Evaluation of the website

Question number	Question	Please circle one answer				
**5.**	Learning to provide exercises using the website was easy for me.	Strongly disagree [0]	Moderately disagree [1]	Neither agree or disagree [2]	Moderately agree [3]	Strongly agree [4]
**6.**	The procedure of signing the stroke survivors up to the website was straight forward.	Strongly disagree [0]	Moderately disagree [1]	Neither agree or disagree [2]	Moderately agree [3]	Strongly agree [4]

**7.**	The procedure of setting the treatment plan up was straight forward.	Strongly disagree [0]	Moderately disagree [1]	Neither agree or disagree [2]	Moderately agree [3]	Strongly agree [4]

Section 3: Please answer the following questions and add your comments, if appropriate:

8. From a physiotherapist's perspective what do you think are the advantages/disadvantages of providing the exercise programme using the website?

Advantages:

Disadvantages:

9. On average, how many times each week did you monitor the exercise programmes?

Once per week [0] ◯ Twice per week [1] ◯ 3-5 times per week [2] ◯ Other [3], please specify:……………..
