# Supplementary material for: Personalised Online Upper-Limb Physiotherapy for Stroke Survivors during the Inpatient Phase: A Feasibility Study
Source: Healthcare (Basel). 2023 Sep 19;11(18):2582. doi: 10.3390/healthcare11182582 (PMC10531470; doi:10.3390/healthcare11182582)
Supplement: Supplementary file 1 [file healthcare-11-02582-s001.zip › Stroke survivors' questionnaire .pdf]

1. **Did you do any of the exercises in your programme?** **Yes [0]** ◯ **No [1]** ◯

If you answered 'Yes', please go straight to Question 2.

If you answered 'No', could you please let us know why that was?

Enter your comments here:

(Thanks for your feedback – there is no need to answer further questions)

Section 1: Evaluation of the augmented exercise programme:

Question number	Question	Please circle one answer				
**2.**	I feel I benefited from the exercise programme.	Strongly disagree [0]	Moderately disagree [1]	Neither agree or disagree [2]	Moderately agree [3]	Strongly agree [4]
**3.**	The exercises were clear and understandable.	Strongly disagree [0]	Moderately disagree [1]	Neither agree or disagree [2]	Moderately agree [3]	Strongly agree [4]
**4.**	The exercise programme did not increase my fatigue (tiredness).	Strongly disagree [0]	Moderately disagree [1]	Neither agree or disagree [2]	Moderately agree [3]	Strongly agree [4]
**5.**	It was easy to contact the physios to make changes to my exercise programme.	Strongly disagree [0]	Moderately disagree [1]	Neither agree or disagree [2]	Moderately agree [3]	Strongly agree [4]
**6.**	I was happy with the length of time it took for the study assessments.	Strongly disagree [0]	Moderately disagree [1]	Neither agree or disagree [2]	Moderately agree [3]	Strongly agree [4]
**7.**	I would be happy to do exercises using this website again in the future.	Strongly disagree [0]	Moderately disagree [1]	Neither agree or disagree [2]	Moderately agree [3]	Strongly agree [4]

Section 2: Evaluation of the website

Question number	Question	Please circle one answer				
**8.**	Doing my exercises through the website gave me the chance to choose when to exercise.	Strongly disagree [0]	Moderately disagree [1]	Neither agree or disagree [2]	Moderately agree [3]	Strongly agree [4]
**9.**	Doing my exercises through the website gave me the feeling of being independent in exercising.	Strongly disagree [0]	Moderately disagree [1]	Neither agree or disagree [2]	Moderately agree [3]	Strongly agree [4]
**10.**	Learning to use the website for my exercises was easy for me.	Strongly disagree [0]	Moderately disagree [1]	Neither agree or disagree [2]	Moderately agree [3]	Strongly agree [4]

Section 3: Please answer the following questions and add your comments, if appropriate:

11. On average, how many times each week did you do your exercise?

Once per week [0] ◯ Twice per week [1] ◯ 3-5 times per week [2] ◯ Other [3], please specify:…………

12. On average, how long did you spend doing the exercises each exercise session?

Less than 30 minutes [0] ◯ Up to 1 hour [1] ◯ 1-2 hours [2] ◯ Other [3], please specify:………………

13. Did your partner/relative help you do your exercise programme? Yes [0] ◯ No [1] ◯ **If yes, please answer the following questions:**

How often? Once per week [0] ◯ Twice per week [1] ◯ 3-5 times per week [2] ◯ Other [3], please specify:…………

What did you need your partner/relative to help you with?

14. Did you ask staff to help you do your exercise programme? Yes [0] ◯ No [1] ◯ **If yes, please answer the following questions:**

How often? Once per week [0] ◯ Twice per week [1] ◯ 3-5 times per week [2] ◯ Other [3], please specify:…………

Who did you ask? Nursing staff [0] ◯ Physiotherapy staff [1] ◯ Other [2], please specify:…………

What did you need the staff to help you with?

15. How difficult/easy was exercising using the website without supervision? Easy [0]◯ Neither easy nor difficult [1]◯ Difficult [2] ◯

Please provide details. For example, did you worry about not exercising properly or knowing how many exercises you needed to do?

Please tell us about things that went well? What was difficult (if anything)?

16. Only for participants who have problems with speech, memory and thinking (aphasia)

Was the website aphasia version helpful?

Certainly yes [0] ◯ To a large extent [1] ◯ To some extent [2] ◯ No [3] ◯ I didn't use it [4] ◯

17. Did you discuss your exercises with other patients? Yes [0] ◯ No [1] ◯

18. Did other patients ask you about your exercises? Yes [0] ◯ No [1] ◯
