# Supplementary material for: Personalised Online Upper-Limb Physiotherapy for Stroke Survivors during the Inpatient Phase: A Feasibility Study
Source: Healthcare (Basel). 2023 Sep 19;11(18):2582. doi: 10.3390/healthcare11182582 (PMC10531470; doi:10.3390/healthcare11182582)
Supplement: Supplementary file 1 [file healthcare-11-02582-s001.zip › TIDieR checklist for the online physiotherapy programme.pdf]

Completed Template for Intervention Description and Replication (TIDieR) checklist for the online physiotherapy programme

1.	Name of the intervention	Augmented upper-limb task-specific exercises for stroke survivors during their hospital stay
2.	Why (rationale and essential elements of the intervention)	**Rationale**<br>• Stroke survivors undergo less rehabilitation than is recommended during their hospital stay; therefore, stroke survivors need to practise augmented rehabilitation.<br>• The available literature on rehabilitation, neuroscience and skill acquisition suggests that a high dose of task-specific practice is needed to facilitate functional recovery after stroke.<br>• It is recommended that stroke survivors practise a high dose of task-specific exercises early after stroke, when neuroplasticity is at its peak, in order to facilitate their functional recovery.<br>• The augmented exercises need to include activities that are functionally relevant and meaningful to stroke survivors in order to facilitate skill acquisition.<br>• The available research on delivering unsupervised upper-limb intervention to stroke survivors during their hospital stay is limited and needs to be strengthened.<br>• In additional, none of the available research used the internet as a tool to deliver unsupervised upper-limb intervention to hospitalised stroke survivors; therefore, studies in this area are required.<br>**Essential elements:**<br>• Progressive task-specific exercises that are prescribed based on stroke survivors' functional capabilities as judged by physiotherapists.<br>• Functionally relevant tasks that are meaningful to each stroke patient.
3.	What (materials)	• The online rehabilitation platform (www.giraffehealth.com) includes three main sections: the home page, a video library of exercises and an advice section that includes generic information about strokes.<br>• The video library includes over 270 diverse exercises, including clips of pre-defined task-specific exercises (such as picking up small objects, lifting a cup and reaching) as well as stretching and passive exercises. The included exercises range in their difficulty.

		• Within each video clip, there is a box that is used to record the website users' performed exercises, the diary section. The diary section is where the website users can find the exercises that they have done and where the website users are able to leave a note about each exercise they attempt.
4.	What (procedures)	• The participant's physiotherapist prescribes/provides an individualised upper-limb exercise programme based on clinical assessment, their goals and level of upper-limb function.<br>• The duration and intensity of the augmented programme are based on the participant's level of upper-limb functional abilities, and this is judged by their physiotherapists.<br>• Each participant is provided with individualised log in details and also provided with explanations of how to use the website in order to access his/her exercises and educational section.<br>• Where appropriate, an explanation of the participant's rehabilitation programme and how to access the website is also provided to carers to support participants with communication difficulties to undertake their rehabilitation programme.<br>• Review the rehabilitation programme once a week (or more if the participants find the programme too easy or too difficult) for four weeks (or before discharge, if earlier).<br>• Final assessments.<br>• Amendments to exercise programme can take place when appropriate based on the participant's response to the exercise programme.<br>• Participants are provided with a tablet and internet access for the duration of the study if they are unable to use their own tablet/laptop to access the website.
5.	Who provided	NHS Physiotherapists.
6.	How (mode of delivery)	• Participants practise their exercise programme independently.<br>• If the participants experience any difficulties, they can contact the researcher (to ask any questions related to the website) and the physiotherapists (to request a change in their programme).<br>• The exercise programme is reviewed once a week by physiotherapists.
7.	Where (location)	The augmented intervention was delivered in the acute stroke unit.

8.	When and how much	**When:**<br>• Once the participant is stable after stroke onset.<br>**Dose:**<br>• Five sessions per week, each lasting 30 minutes, for a period of 4 weeks in addition to usual rehabilitation care.<br>• Participants with low exercise capacity may begin with a smaller number of sessions and/or shorter overall time of each session and vice versa.<br>• Each exercise programme is prescribed by physiotherapists based on participants' level of functional ability.
9.	Tailoring	• Physiotherapists prescribed the augmented upper-limb exercise programmes to individuals by assessing their upper-limb functional level as well as considering their goals.<br>• Physiotherapists progress the level of exercise difficulty considering individuals' response to the augmented intervention.
10.	How well	**Planned:**<br>Participants were asked to use the diary section (by ticking the box) each time they completed an exercise to record their practised exercises. For participants who required support to use the website, carers were asked to use the diary section every time the participant completed an exercise.<br><br>**Adherence to the augmented intervention was recorded by participants and/or carers using the diary section.**<br>The number of completed exercise diaries each week and over the intervention period (up to four weeks) is used to measure adherence. Adherence was presented as a percentage of completed exercise diaries based on the participant's prescribed rehabilitation programme.
